# A-kinase anchor protein 4 (AKAP4) a promising therapeutic target of colorectal cancer

**DOI:** 10.1186/s13046-015-0258-y

**Published:** 2015-11-21

**Authors:** Nirmala Jagadish, Deepak Parashar, Namita Gupta, Sumit Agarwal, Sapna Purohit, Vikash Kumar, Aditi Sharma, Rukhsar Fatima, Amos Prashant Topno, Chandrima Shaha, Anil Suri

**Affiliations:** Cancer Microarray, Genes and Proteins Laboratory, National Institute of Immunology, Aruna Asaf Ali Marg, New Delhi, 110 067 India; Cell Death and Differentiation Research Laboratory, National Institute of Immunology, Aruna Asaf Ali Marg, New Delhi, 110 067 India

**Keywords:** Cancer testis antigens, Gene silencing, Therapeutic target, AKAP4

## Abstract

**Background:**

Colorectal cancer (CRC) ranks third among the estimated cancer cases and cancer related mortalities in the Western world. Early detection and efficient therapy of CRC remains a major health challenge. Therefore, there is a need to identify novel tumor markers for early diagnosis and treatment of CRC.

**Methods:**

A-kinase anchor protein 4 (AKAP4) gene and protein expression was monitored by quantitative polymerase chain reaction (qPCR), reverse transcription (RT)-PCR and Western blotting in normal colon tissue lysate, normal colon epithelial cells and in colon cancer cell lines viz., Caco-2, COLO205, COLO320DM, HCT-15, HCT116, HT-29, SW480, and SW620. The effect of AKAP4 on cellular growth, migration and invasion abilities was studied using gene silencing approach. The role of AKAP4 in various pathways involved in cell cycle, apoptosis, senescence was investigated in *in vitro* and in human xenograft mouse model.

**Results:**

Our studies showed that AKAP4 gene and protein expression was expressed in all colon cancer cells while no expression was detectable in normal colon cells. Ablation of AKAP4 led to reduced cellular growth, migration, invasion and increased apoptosis and senescence of CRC cells in *in vitro* assays and tumor growth in human xenograft mouse. Human colon xenograft studies showed a significant decrease in the levels of cyclins B1, D and E and cyclin dependent kinases such as CDK1, CDK2, CDK4 and CDK6. Interestingly, an up-regulation in the levels of p16 and p21 was also observed. Besides, an increase in the levels of pro-apoptotic molecules AIF, APAF1, BAD, BID, BAK, BAX, PARP1, NOXA, PUMA and cyt-C and Caspase 3, 7, 8 and 9 was also found in cancer cells as well as in xenograft tissue sections. However, anti-apoptotic molecules BCL2, Bcl-x_L_, cIAP2, XIAP, Axin2 and Survivin were down regulated in these samples. Our data also revealed elevated expression of epithelial marker E-cadherin and down regulation of EMT markers N-cadherin, P-cadherin, SLUG, α-SMA, SNAIL, TWIST and Vimentin. Further ablation of AKAP4 resulted in the down regulation of invasion molecules matrix metalloproteinase MMP2, MMP3 and MMP9.

**Conclusion:**

AKAP4 appears to be a novel CRC-associated antigen with a potential for developing as a new clinical therapeutic target.

**Electronic supplementary material:**

The online version of this article (doi:10.1186/s13046-015-0258-y) contains supplementary material, which is available to authorized users.

## Background

Colorectal cancer (CRC) is the third common cancer and a common cause of cancer-related death among US men and women [1]. CRC progresses through multi-step process at both genotypic and phenotypic level [2]. Since the disease is diagnosed at late stages, the treatment options are limited for CRC patients [3]. Cancer testis (CT) antigens are unique class of tumor restricted antigens which have been studied in various malignancies and have been shown to be associated with tumor growth [4]. Recently, CT antigen SPAG9 expression has been shown to be associated with CRC [5]. Although CT antigens are the core focus in the development and clinical testing of experimental therapeutic targets, their involvement at molecular level in cell cycle regulation, senescence, apoptosis, epithelial mesenchymal transition have not yet fully understood.

Our previous studies have demonstrated an association of a novel CT antigen A-kinase anchor protein 4 (AKAP4) expression in breast cancer [6], cervical cancer [7, 8] and in ovarian cancer [9]. More recently, we have demonstrated AKAP4 expression in majority of colorectal cancer (CRC) tissue specimens and did not find AKAP4 expression in normal colon tissue specimens [10]. Our data suggested that AKAP4 could be playing a potential role in various malignant properties of CRC. Cell cycle deregulation, resistance to cell death, increased cell invasion and migration potential are some important hallmarks of cancer [11]. Recent studies have shown that ablation of CT antigens in different cancers lead to cell cycle arrest and senescence [12], apoptosis [13] and inhibition in cell migration and invasion [14]. However, till date the role of CT antigens have not been investigated in cell cycle, senescence, apoptosis and epithelial mesenchymal transition (EMT) in CRC cases.

In the present study, we analyzed the expression of AKAP4 gene and protein in CRC cell lines and its potential role in cellular growth, proliferation, migration and invasion at molecular level in *in vitro* and *in vivo* in human CRC xenograft mouse model. We show that ablation of AKAP4 lead to the down regulation of cyclins (Cyclin B1, Cyclin D1 and Cyclin E) along with their CDK-partners (CDK1, CDK2, CDK4 and CDK6) and upregulation of cyclin dependent kinase inhibitors (CKIs), p16, p21 and retinoblastoma. Further, we investigated its role in cellular proliferation, migration, invasion, wound healing, colony forming abilities and tumor growth which suggested that AKAP4 could be used as a novel therapeutic target for CRC treatment.

## Methods

### Cell culture

Human colon cancer cell lines COLO 205 and HCT 116 were procured from the American Type Culture Collection (ATCC, Manassas, VA) and were maintained according to standard procedures. Human colon cancer cell lines CaCo-2, COLO320 DM, HCT-15, HT-29, SW480 and SW620 were procured from National Centre for Cell Sciences (NCCS, Pune, Maharashtra, India), and were used within 8 weeks by growing in DMEM medium (Invitrogen Life Technologies, Carlsbad, CA, USA) supplemented with 10 % fetal bovine serum (FBS) maintained in a humidified 37 °C and 5 % CO_2_ incubator and were checked for mycoplasma contamination by mycoplasma PCR detection kit (Applied Biological Materials Inc., Richmond, Canada). Human normal colon epithelial cell NCM460 was procured and maintained according to manufacturer’s directions (INCELL Corporation LLC, Saint Antonio, Texas, USA). Transient transfection was carried out by seeding 1 × 10^5^COLO 205 or HCT 116 cells in 6-well plate using Lipofectamine reagent (Invitrogen, Life Technologies, Carlsbad, CA) according to the manufacturer’s instructions.

### Antibodies

Western blot and immunohistochemistry analysis was carried out using following antibodies; mouse anti-AKAP4 antibody was procured from Sigma-Aldrich (St. Louis, MO, USA), mouse anti-proliferating cell nuclear antigen (PCNA), mouse anti-calnexin (endoplasmic reticulum maker), mouse anti-GM130 (Golgi body marker) and mouse anti-lamin A/C (nuclear envelope marker) were purchased from Santa Cruz Biotechnology, USA. Horseradish peroxidase-conjugated anti-rat IgG, FITC-conjugated anti-rat IgG, and Texas Red-conjugated anti-mouse IgG were procured from Jackson ImmunoResearch Laboratories, West Grove, PA, USA. Mouse anti-beta actin, anti-MTCO2 (mitochondrial marker), mouse anti-E-cadherin, mouse anti-N-cadherin, mouse anti-P-cadherin, Matrix metalloproteinases (MMP’s): rabbit anti-MMP2, rabbit anti-MMP3, mouse anti-MMP9, rabbit anti-SNAIL, mouse anti-SLUG, mouse anti-TWIST, mouse anti-alpha smooth muscle actin (αSMA), rabbit anti-Vimentin, mouse anti-Caspase 3, mouse anti-AIF, rabbit anti-APAF1, rabbit anti-XIAP, rabbit anti-Survivin, rabbit anti-DCR2, mouse anti-CDK1,rabbit anti-CDK2, and rabbit anti-phosphoRb were procured from Abcam, Cambridge, UK. Mouse anti-BCL-2-associated death promoter (BAD), rabbit anti-BCL-2 homologous antagonist/killer (BAK), mouse anti-BCL-2-associated X Protein (BAX), rabbit anti-BID, rabbit anti-Bcl-x_L_, mouse anti-cytochrome-C, mouse anti-NOXA, rabbit anti-p53 upregulated modulator of apoptosis (PUMA), mouse anti-poly ADP ribose Polymerase 1 (PARP1), mouse anti-Caspase 7, mouse anti-Caspase 8, mouse anti-Caspase9, rabbit anti-cIAP2, Cyclin-dependent kinases (CDKs): mouse anti-CDK4 and mouse anti-CDK6, mouse anti-Cyclin B1, mouse anti-Cyclin D1, mouse anti-Cyclin E, anti-cyclin-dependent kinase inhibitor (CKI), mouse anti-p21, mouse anti-p16, and mouse anti-Retinoblastoma (Rb) were procured from Santa Cruz Biotechnology. Mouse anti-B-cell lymphoma 2 (BCL-2) was procured from Cell Signaling Technology, USA.

### Reverse transcription-polymerase chain reaction (RT-PCR) and quantitative PCR (qPCR)

Total RNA from all cancer cell lines and normal colon cells was isolated using RNeasy Mini kit (Qiagen GmbH, Hilden, Germany) as per manufacturer’s protocol. The RNA was reverse transcribed using a set of primers and High-Capacity cDNA Reverse Transcription Kit (Applied Biosystems, Carlsbad, CA) as described earlier [8]. Following AKAP4 specific primers were used: *AKAP4* Forward primer 5′-*TGATACTACAATGATGTCTGATGAT*-3′, *AKAP4* Reverse primer 5′-*GGAACTAGCAGCATCCTTGTAATCTTTATC*-3′, *β-actin* was used as an internal control to check the quality of cDNA synthesis with following primers: *β-actin* Forward primer 5′-*ATCTGGCACCACACCTTCTACAATGAGCTGCG*-3′, *β-actin* Reverse primer 5′-*CGTCATACTCCTGCTTGCTGATCCACATCTGC*-3′. The PCR products were electrophoresed on 2 % agarose gel and photographed under UV light in EC3 Imaging System (UVP, Upland, CA). The amplicons of AKAP4 thus obtained were sub-cloned into TOPO vector (Invitrogen, Carlsbad, CA) to confirm the sequence of the *AKAP4. β-Actin* mRNA expression was checked as an internal control. Quantitative PCR was done using 5 ng of cDNA from normal colon epithelial cells and eight colon cancer cell lines in triplicate with Brilliant III Ultra-Fast SYBR QPCR MM (Agilent Technology, USA) in iCycleriQmulticolor real time PCR detection system (Bio-Rad, CA, USA) according to manufacturer’s instructions. *β-Actin* was used as an internal control in all the reactions. *AKAP4* mRNA expression was also checked in normal colon epithelial cells as a negative control. *AKAP4* gene expression levels was subsequently normalized using expression levels of endogenous control *β-Actin*in the same mRNA samples in each colon cancer cell lines.

### Western blotting, flow cytometric analysis and indirect immunofluorescence

Proteins from colorectal cancer cell extracts (10 μg/lane) and from normal colon tissue were resolved on 10 % sodium dodecylsulphate-10 % Polyacrylamide gel electrophoresis (SDS-PAGE) and Western blotting was carried out as described earlier [8]. The protein was transferred onto the polyvinylidene fluoride (PVDF) membrane (Millipore Corporation, USA). Briefly, Western blotting was carried out employing mouse monoclonal AKAP4 antibody (Sigma-Aldrich, St. Louis, MO) and goat anti-mouse IgG Horseradish Peroxidase (HRP) (Jackson Immuno Research Laboratories, Inc., Baltimore, USA). Immunoreactivity against AKAP4 protein was developed by Immobilon Western Chemiluminescent HRP substrate (Millipore Corporation, USA).

Flow cytometeric analysis was carried by culturing CRC cells. Subsequently cells were harvested and processed for AKAP4 surface localization as described earlier [8] using anti-AKAP4 antibody or control IgG followed by goat anti-mouse IgG fluorescein isothiocyanate (FITC) conjugate (Jackson Immuno Research Laboratories, Inc., Baltimore, USA) as secondary antibody. The flow cytometric analysis was done in a flow cytometer (BD-CALIBUR model; BD Biosciences, California, USA). Data acquisition and analysis was done using Cell QuestPro software.

Indirect immunofluorescence was carried in CRC cells by probing with anti-AKAP4 antibody or a control IgG as described earlier [8]. The cells were subsequently incubated with FITC conjugated goat anti-mouse IgG. The slides were washed and mounted in antifade reagent (Invitrogen Life Technologies Corporation, USA). AKAP4 protein co-localization was studied as described earlier [8]. Briefly, cells were incubated with different reagents using endoplasmic reticulum marker (calnexin, 6D195, sc-70481; Santa Cruz Biotechnology, Santa Cruz, CA), golgi bodies marker (GM130 B-10; Santa Cruz Biotechnology), mitochondria marker (MTCO2; Abcam) and nuclear envelope marker (lamin A/C 636; Santa Cruz Biotechnology). Texas red conjugated anti-mouse IgG was used as secondary antibody for co-localization. Photo micrographs were captured using the Carl Zeiss LSM 510 Meta confocal microscope (Germany) in central confocal microscopy facility.

### Short hairpin RNA silencing of *AKAP4* gene

Plasmid driven short hairpin RNA (shRNA) constructs and NC shRNA (scrambled shRNA) were procured from Super Array (Frederick, MD, USA). The following target sequences were used in this study: *AKAP4*: 5′-TCTATGTTCACTTGATCGG-3′ (AKAP4 shRNA1, Clone ID V2LHS-53112); 5′-CAAGCGAACGGGCAATTTA-3′ (AKAP4 shRNA2 Clone ID V2LHS-53113); 5′-TTACCAGAGAAGATAGTCG-3′ (AKAP4 shRNA3 Clone ID V2LHS-53116) and 5′-ATCTCGCTTGGGCGAGAGTAAG-3′ (NC shRNA, RHS4430-99147765). The shRNA plasmids were prepared and transfected in COLO 205 and HCT 116 cells using lipofectamine and plus reagent (Invitrogen Life Technologies Corporation, USA). Further, qPCR was carried out to find out the knockdown of *AKAP4* mRNA with various targets under investigation. Total RNA was extracted using RNeasy mini kit (Qiagen, Germany) and subsequently subjected to synthesize cDNA using High Capacity cDNA Reverse Transcription Kits (Applied Biosystems, USA). Quantification of *AKAP4* mRNA was done using 5 ng of cDNA employing following AKAP4 specific primers (Forward primer 5′-*TGATACTACAATGATGTCTGATGAT*-3′ and Reverse primer 5′-*GGAACTAGCAGCATCCTTGTAATCTTTATC*-3′) and iQ SYBR Green Supermix (Bio-Rad Corporation Inc., California, USA). Cell lysates post 48 h post transfection were prepared for Western blotting as described earlier [8].

### Cell viability and cellular proliferation assay

Viability assay was carried out by transfecting AKAP4 shRNA3 in COLO 205 and HCT 116 cells and were visualized using chromogenic dye 3-(4,5-dimethylthiazol-2-yl)-2, 5-diphenyltetrazolium bromide (MTT, Sigma-Aldrich, St. Louis, MO). Absorbance at 570 nm-650 nm was recorded on ELISA plate reader (Molecular Devices, Sunnyvale, CA). In addition, cellular growth analysis was also carried out as described earlier [8]. Cells after transfection with AKAP4 shRNA3 or NC shRNA were counted at 24 h, 48 h and 72 h. The experiments were repeated twice in triplicates.

### Colonogenic assay

Both COLO 205 and HCT 116 cells after transfection with AKAP4 shRNA3 or NC shRNA targets were seeded in 6-well plates at three different cell densities in duplicates (400, 800 and 1200). Ten days post-seeding, the cells were fixed with 5 % glutaraldehyde in phosphate buffered saline (PBS) and stained with 0.5 % toluidine blue (Sigma-Aldrich, St. Louis, MO). The colonies were manually counted after washing cells with PBS. Images of representative fields were also captured using Nikon Eclipse E 400 microscope (Nikon, Fukok, Japan). Each experiment was repeated twice in triplicates.

### TUNEL assay

The effect of shRNA treatment in cancer cells on DNA fragmentation was assessed using Apo-BrdU- Red *in-situ* DNA fragmentation assay kit (Biovision, K404-60). AKAP4 shRNA3or NC shRNA transfected COLO 205 and HCT 116 cells were harvested by trypsinization and processed as per manufacturer’s instructions. The cells were analyzed at 576 nm using BD-FACS VERSA. (BD Biosciences, California, USA).

### AnnexinV-PerCP-Cy5-5-A

To study the effect of AKAP4 shRNA3or NC shRNA on apoptosis, cells transfected with shRNA were stained by annexin V using annexinV-PerCP-Cy5-5-A staining kit (Biovision). Staining was performed according to the manufacturer’s instructions. The cells were analyzed with a flow cytometer (BD-CALIBUR model; BD Biosciences, California, USA). Data acquisition and analysis was done using Cell QuestPro software.

### Cellular senescence assay

Both COLO 205 and HCT 116 cells at a density of 3 x 10^5^each were transfected with AKAP4 shRNA3 or NC shRNA in 6-well plate. Post 48 h transfection, senescence assay was carried out using Senescence kit (Sigma-Aldrich, St. Louis, MO, USA) as per manufacturer’s protocol. The images were captured using Nikon Eclipse E 400 microscope (Nikon, Fukok, Japan).

### Cell invasion and migration

To investigate the potential role of AKAP4 in cellular migration and invasion assay were performed as described earlier [8].

### Scanning electron microscopy

COLO 205 and HCT 116 cells were transfected with AKAP4 shRNA3or NC shRNA. Cells were seeded onto 12 mm coverslip and were fixed with 2.5 % glutaraldehyde in 4 % paraformaldehyde solution in 0.2 M sodium cacodylate at different time intervals (24 h and 48 h). Subsequently, cancer cells were processed by washing with sodium cacodylate solution, followed by staining with osmium tetraoxide. Coverslip having cells were washed with deionised water and dehydrated using different gradients of methanol (25–100 %). Critical point drying was done with hexa methyl disilane (HMDS) and coverslips were mounted on aluminium stubs. Gold and palladium coating was done using electron sputter coater under vacuum by Argon based Thermionic emission. The images were captured using electron microscope (EVO LSM10 Zeiss, Germay) at 20 kV using SmartSEM software in central microscopic facility.

### *In-vivo* xenograft studies

Human tumor xenografts were established in 6–8 weeks old athymic nude mice (NII, NIH [S]nu/nu) as described earlier [8]. All investigations in animals were carried out after obtaining ethical clearance from Institute animal ethical committee (IAEC). Intratumor injections of AKAP4 shRNA3 or NC shRNA (first dose of 50 µg followed by 25 µg booster doses) were initiated when the tumor volume was ~50-100 mm^3^ as described earlier [8]. Mice were sacrificed after 49 days and tumors were excised, weighed and processed for IHC for AKAP4, PCNA and for various molecules of cell cycle, apoptosis and EMT pathway.

### Immunohistochemistry (IHC)

Immunohistochemical analysis was performed on 4-μm thick sections of tumor tissue excised from AKAP4 shRNA3or NC shRNA treated mice as described earlier [8]. Briefly, sections were deparaffinized, rehydrated, washed with phosphate buffer saline (PBS; pH7.2) and were incubated in methanolic H_2_O_2_ (9:1) for 45 min to block and remove all traces of endogenous peroxidase. Subsequently, tissue sections were blocked with 5 % normal goat serum for 1 h at RT and probed with various antibodies (anti-AKAP4, anti-PCNA,anti-p16, anti-p21, anti-CDK1, anti-CDK2, anti-CDK4, anti-CDK6, anti-CyclinD1,anti-Cyclin B1,anti-Cyclin E, anti-AIF, anti-APAF1, anti-BAD, anti-BID, anti-BAK, anti-BAX, anti-PARP1, anti-PUMA, anti-NOXA, anti-cyt-C, anti-caspase 3, anti-caspase 7, anti-caspase 8, anti-caspase9, anti-BCL-2,anti-Bcl-x_L_,anti-cIAP2, anti-XIAP, anti-Survivin, anti-E-cadherin, anti-N-cadherin, anti-αSMA, anti-SNAIL, anti-TWIST, anti-Vimentin, anti-MMP2 and anti-MMP9 antibodies) for overnight at 4 °C. After three washes with PBS, sections were incubated with secondary antibody (HRP-conjugated goat anti-rat IgG or HRP-conjugated goat anti-mouse IgG or HRP-conjugated donkey anti-rabbit IgG; Jackson Immuno-Research Laboratories, West Grove, PA). After incubation sections were subjected to three washings with PBS and the color was developed using 3,3′-Diaminobenzidine (Sigma- Aldrich, St. Louis, MO) as a substrate. Slides were counter stained with hematoxylin solution, mounted and observed under a Nikon Eclipse E400 microscope (Nikon, Fukuoka, Japan).

## Results

### *AKAP4* gene and protein expression in CRC cell lines

**Fig. 1:**
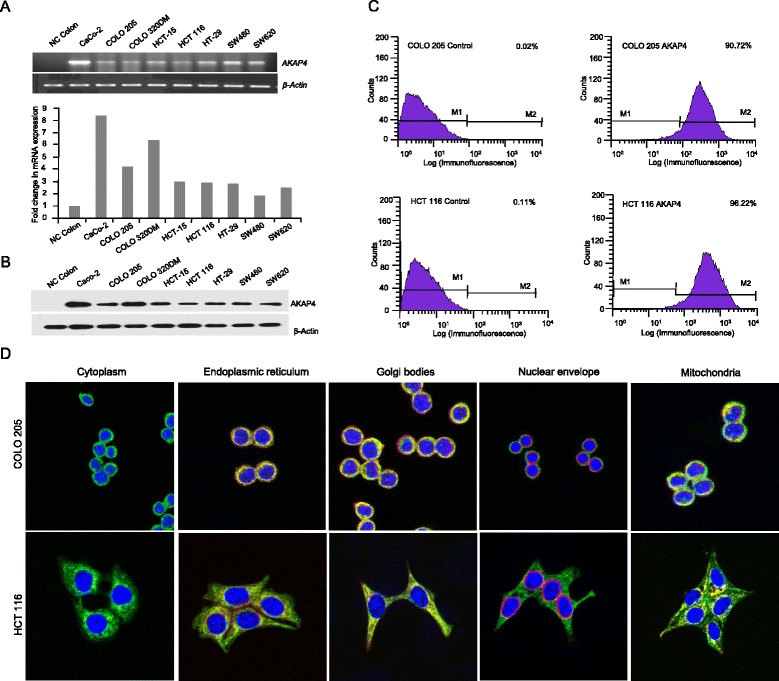
AKAP4 gene and protein expression. **a** RT-PCR and qPCR analysis shows *AKAP4* mRNA expression in various CRC cell lines along with normal colon (NC colon). **b** Western blot analysis demonstrates AKAP4 protein expression in various CRC cell lines and in normal colon cells (NC colon). **c** Flow cytometric analysis reveals AKAP4 expression in COLO 205 and HCT 116. **d** Indirect immunofluorescence and co-localization studies show the cytoplasmic localization and co-localization (orange-yellowish staining) of AKAP4 in endoplasmic reticulum, Golgi bodies and mitochondria in COLO 205 and HCT 116 CRC cells, however no co-localization was observed in nuclear envelope

We investigated the association of AKAP4 with various malignant properties of CRC cells by carrying out *in-vitro* assays and in *in-vivo* colorectal human xenograft model. Initially, we examined COLO 205 and HCT 116 cells for the *AKAP4* gene expression by RT-PCR using *AKAP4* specific primers. As shown in Fig. 1a, we observed the *AKAP4* gene expression in both CRC cell lines. We further assessed *AKAP4* mRNA expression in normal colon epithelial cells, CaCo-2, COLO 205, COLO320DM, HCT-15, HT-29, SW480 and SW620 colon cancer cell lines by quantitative PCR (qPCR). All cancer cell lines showed higher levels of AKAP4 expression compared to normal colon epithelial cells (Fig. 1a). *AKAP4* expression was 8.3 fold higher in CaCo-2, 4.2 fold in COLO 205, 6.4 fold in COLO 320, 3.1 fold in HCT-15, 2.9 fold in HCT 116, 2.8 folds in HT-29, 1.8 fold in SW 480 and 2.4 fold higher in SW 620 as compared to normal colon epithelial cells.

We further evaluated the AKAP4 protein expression in CRC cell lines by Western blotting and flow cytometry. As shown in Fig. 1b, AKAP4 immuno-reactivity was found in COLO 205 and HCT 116 CRC cells. Further FACS analysis of these cells (Fig. 1c) revealed surface localization of AKAP4 protein in COLO 205 (90.72 %) and HCT 116 (96.22 %) cells as compared to unstained controls (Fig. 1c). Confocal microscopy images showed a cytoplasmic distribution of AKAP4 protein in both COLO 205 and HCT 116 (Fig. 1d) with prominent localization in endoplasmic reticulum, mitochondria and Golgi bodies. Notably, AKAP4 did not localize with nuclear envelop. Since both the cell lines exhibited similar expression profile and intracellular localization of AKAP4, we used COLO 205 and HCT 116 cell lines in all our subsequent studies.

### Hairpin driven gene silencing ablates **AKAP4** protein expression

**Fig. 2:**
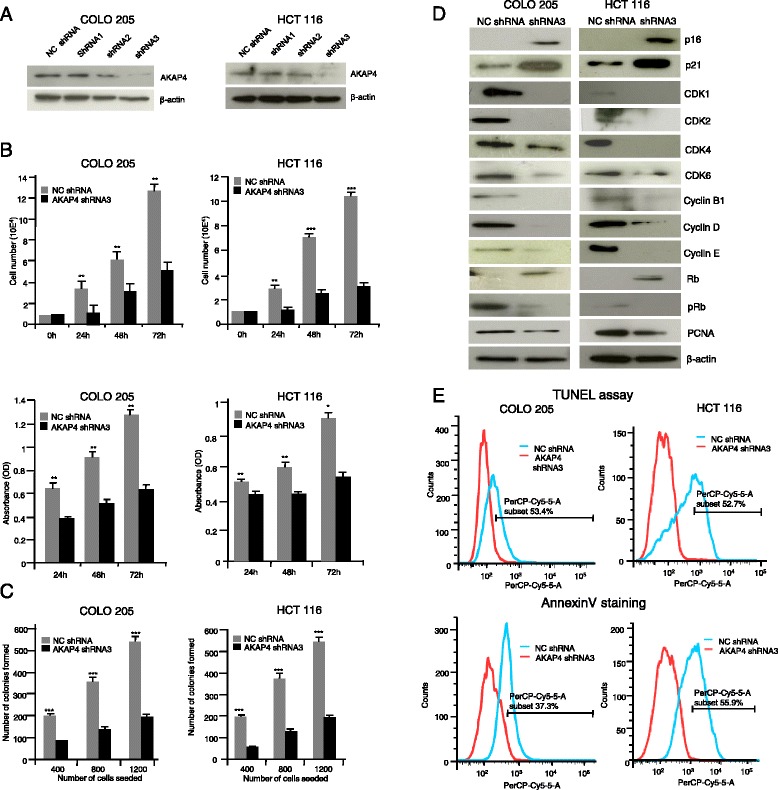
Ablation of AKAP4 protein alters malignant properties of CRC cells. **a** Western blot show knockdown efficiency of shRNA targets against AKAP4 protein in COLO 205 and HCT 116 cells. **b** Histograms depict the effect of AKAP4 shRNA3 on cellular proliferation and cell viability of COLO 205 and HCT 116 cells as compared to NC shRNA at 24 h, 48 h and 72 h. **c** Histogram depicts the difference between the number of colonies being formed in AKAP4 shRNA3 transfected COLO 205 and HCT 116 cells as compared to NC shRNA transfected COLO 205 and HCT 116 cells. **d** The blots show changes in the expression of various molecules that are involved at different phases of the cell cycle in COLO 205 and HCT 116 cells when transfected with AKAP4 shRNA3 as compared to NC shRNA. Upregulation of p16, p21, Rb protein while down regulation of Cyclins namely Cyclin B1, D, E and cyclin dependent kinases (CDK’s), CDK 1, 2, 4, 6 and phosphorylated Rb was observed. Proliferation marker PCNA was also down regulated. β-actin was used as a loading control. **e** Flow cytometric analysis reveals DNA fragmentation as assessed by TUNEL assay. Surface expression of phosphotidyl serine was assessed by AnnexinV-PerCP-Cy5-5-A staining. The blue peak shows the cells transfected with NC shRNA while the red peak shows the cells transfected with AKAP4 shRNA3. *P < 0.05, **P < 0.001, ***P < 0.0001

Three shRNA targets against *AKAP4* gene were used to regulate the expression *AKAP4* gene in COLO 205 and HCT 116 cells and were analyzed by RT-PCR. Our analysis revealed 17 %, 8 % and 79 % *AKAP4* gene knockdown by shRNA target 1, 2 and 3 in COLO 205 (Additional file 1: Figure S1A) and 15 %, 12 % and 71 % in HCT 116 (Additional file 1: Figure S1A) respectively relative to NC shRNA. Further, Western blot analysis confirmed the down regulation of AKAP4 protein specifically in the presence of shRNA target 3 (Fig. 2a). As expected the NC shRNA did not knockdown the gene or protein levels. Importantly, the AKAP4 shRNA3 showed a higher efficiency in gene knockdown and protein expression in both COLO 205 and HCT 116 cells (Fig. 2a and Additional file 1: Figure S1A).

### AKAP4 shRNA inhibits cellular growth and colony formation ability

In cellular proliferation assay, knockdown of AKAP4 using AKAP4 shRNA3 inhibited cellular growth of COLO 205 cells by 62.16 %, 46.97 % and 64.29 % at 24 h, 48 h and 72 h respectively (Fig. 2b). Similarly, in HCT 116 cells 55.26 %, 67.44 % and 69.11 % reduction in cellular growth was observed at 24 h, 48 h and 72 h respectively (Fig. 2b). In addition, AKAP4 ablation also reduced the cell viability by 39.39 %, 43.32 % and 49.99 % at 24 h, 48 h and 72 h in COLO 205 cells (Fig. 2b). Similarly, 19.90 %, 27.31 % and 38.40 % reduction was observed in HCT cell viability at 24 h, 48 h and 72 h respectively (Fig. 2b). Besides, the colony forming ability was also reduced by 57–64 % (400–1200 cells) for COLO 205 cells (Fig. 2c) whereas 64–70 % (400–1200 cells) reduction was observed in HCT 116 cells (Fig. 2c). No reduction in cellular proliferation, viability and colony formation was observed in both COLO 205 and HCT 116 cells with NC ShRNA.

**Fig. 3:**
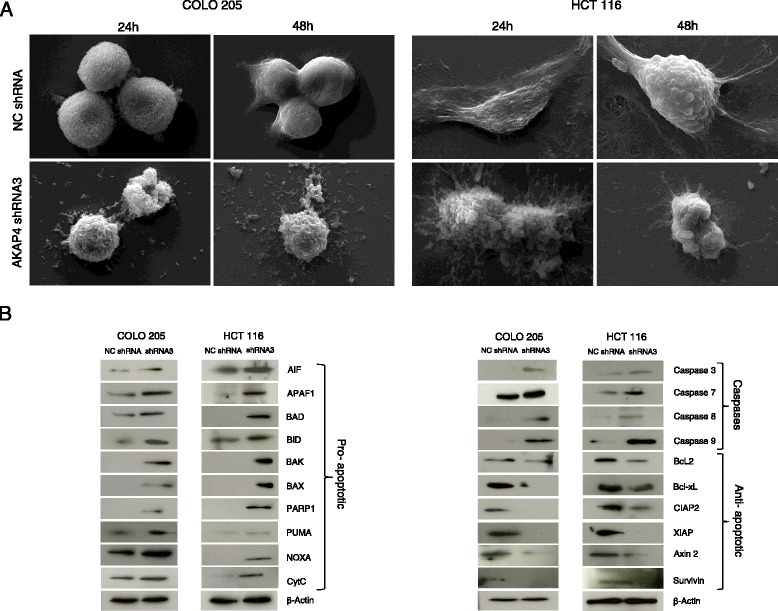
AKAP4 gene and protein silencing initiates apoptosis. **a** The representative scanning electron microscope (SEM) images show changes in phenotypic characteristics of COLO 205 and HCT 116 cells when transfected with AKAP4 shRNA3 as compared to NC shRNA. AKAP4 shRNA3 treatment shows intense membrane blebbing and holes on the surface of cells at 24 h and 48 h as compared to cells when treated with NC shRNA. **b** The blots show changes in the expression of various molecules that are involved in apoptotic pathway in COLO 205 and HCT 116 cells when transfected with AKAP4 shRNA3 as compared to NC shRNA. The results demonstrates up regulation of pro-apoptotic molecules like AIF, APAF1, BAD, BID, BAK, BAX, PARP1, PUMA, NOXA, cyt-C and Caspase 3, Caspase 7, Caspase 8 and Caspase 9, while down regulation of anti-apoptotic molecules like Bcl-x_L_, cIAP2, XIAP, BCL2, Survivin, and Axin 2. β-actin was used as a loading control

We further studied the levels of various molecules involved in cellular proliferation, colony formation and during different phases of cell cycle. Western blot results showed that there was a significant decrease in cyclins (B1, D, and E) and cyclin dependent kinases (CDK1, CDK2, CDK4, and CDK6) in both CRC cell lines (Fig. 2d). Notably, the level of phosphorylated Rb, PCNA were also found down regulated indicating reduction in cellular proliferation (Fig. 3). Interestingly, there was an up-regulation in the levels of p16, p21 and tumor suppressor gene, retinoblastoma (Rb). These results suggest that AKAP4 ablation was associated with cell cycle arrest and inhibition of cellular proliferation of CRC cells.

### Knock down of AKAP4 induces apoptosis in CRC cells

We next investigated the effect of ablation of AKAP4 on apoptosis of both COLO 205 and HCT 116 cells employing TUNEL assay and Annexin V-PerCP-Cy5-5-A staining. TUNEL assay results showed that apoptosis was induced in AKAP4 shRNA3 transfected COLO 205 and HCT 116 cells by53.4 % and 52.7 % respectively (Fig. 2e). Similarly, AnnexinV-PerCP-Cy5-5-Astaining showed that in AKAP4 shRNA3 transfected COLO 205 and HCT 116 cells by 37.33 % and 55.97 % respectively (Fig. 2e). We also investigated the morphological changes during apoptosis in the CRC cells treated with AKAP4 shRNA3 and NC shRNA using scanning electron microscopy (SEM). SEM images were acquired at different time intervals. As shown in Fig. 3a, the SEM photomicrograph revealed significant apoptotic changes in AKAP4 shRNA3 transfected COLO 205 and HCT 116 cells as compared to NC shRNA transfected cells. Blebbing, holes and apoptotic bodies were seen in both COLO 205 and HCT 116 cells post 24 and 48 h of transfection with AKAP4 shRNA3. However, no morphological changes were observed in both CRC cells transfected with NC shRNA. Subsequently, various anti-apoptotic and pro-apoptotic molecules were also investigated in both AKAP4 shRAN3 and NC shRNA treated cells. Our results revealed that pro-apoptotic molecules AIF, APAF1, BAD, BID, BAK, BAX, PARP1, NOXA, PUMA and cyt-C and caspase proteins Caspase 3, 7, 8 and 9 (Fig. 3b) were up-regulated. Further, anti-apoptotic molecules such as BCL2, Bcl-x_L_, cIAP2, XIAP, Axin2 and Survivin were down regulated (Fig. 3b).

### *AKAP4* gene silencing reduces the cellular motility

**Fig. 4:**
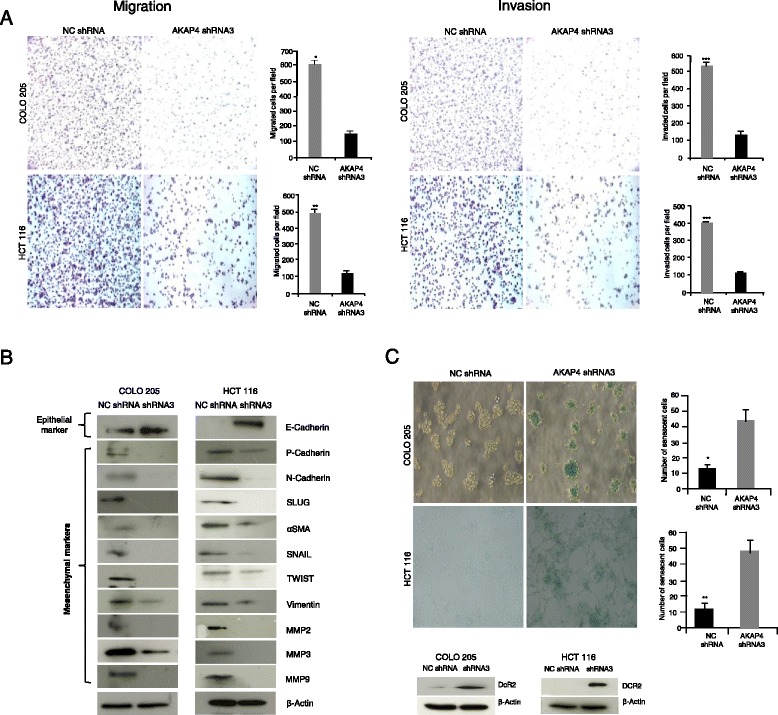
Knockdown of *AKAP4* gene reduces cellular motility and induces cellular senescence in CRC cells. **a** Representative photomicrographs show the reduction in COLO 205 and HCT 116 cells migrating/invading through transwell membrane when transfected with AKAP4 shRNA3 compared to NC shRNA. Histogram depicts significant reduction in number of cells migrating/ invading through insert membrane when transfected with AKAP4 shRNA3 as compared to NC shRNA. **b** Western blot analysis of the molecules of EMT demonstrates up regulation of epithelial marker E-cadherin, while down regulation of mesenchymal markers like P-cadherin, N-cadherin, SLUG, α-SMA, SNAIL, TWIST, Vimentin and MMP 2, 3, 9. β-actin was used as a loading control .**c** Senescence assay: representative phase contrast microscopic images showed higher β-galactosidase activity (green staining) in AKAP4 shRNA3 transfected COLO 205 and HCT 116 cells as compared to NC shRNA transfected cells. Histogram depicts the quantitative difference in the β-galactosidase activity in AKAP4 shRNA3 transfected COLO 205 and HCT 116 cells as compared to NC shRNA transfected cells. Western blot analysis reveals the up-regulation of senescence marker, DCR2, in AKAP4 shRNA3 transfected COLO 205 and HCT 116 cells as compared to NC shRNA transfected cells. *P < 0.05, **P < 0.001, ***P < 0.0001

The effect of AKAP4 knockdown on cellular motility was assessed by carrying out by cell migration and invasion assays. Ablation of AKAP4 protein resulted in 71.17 % and 68.28 % reduction in migration ability of COLO 205 and HCT 116 cells respectively (Fig. 4a). Similarly, a 72.35 % and 67.52 % reduction was observed in invasive abilities of COLO 205 and HCT 116 cells respectively (Fig. 4a). We next investigated the role of various signaling pathways that contributed towards migration and invasion of cells. Our data revealed that expression of epithelial marker E-cadherin was up regulated, whereas epithelial to mesenchymal transition markers, N-cadherin, P-cadherin, SLUG, αSMA, SNAIL, TWIST and Vimentin, were down regulated (Fig. 4b). Interestingly, expression of invasion molecules matrix metalloproteinase MMP2, MMP3 and MMP9 were also down regulated following AKAP4 ablation. These results suggest that AKAP4 may play an important role in cellular motility.

### AKAP4 is associated with cellular senescence

Next, we examined cellular senescence in COLO 205 and HCT 116 cells post AKAP4 knockdown by carrying out β-galactosidase staining (Fig. 4c). The percentage of senescent cells was significantly higher in COLO 205 (44.2 %) and HCT 116 cells (48.4 %) when transfected with AKAP4 shRNA3 as compared to NC shRNA staining (Fig. 4c). AKAP4 ablation also resulted in the upregulation of decoy receptor 2 (DCR2) protein expression (Fig. 4c) which is a marker for cellular senescence. These results indicated the ablation of AKAP4 protein seems to contribute towards the senescent state of cancer cells.

### *AKAP4* down-regulation inhibits colon cancer xenograft in mouse model

**Fig. 5:**
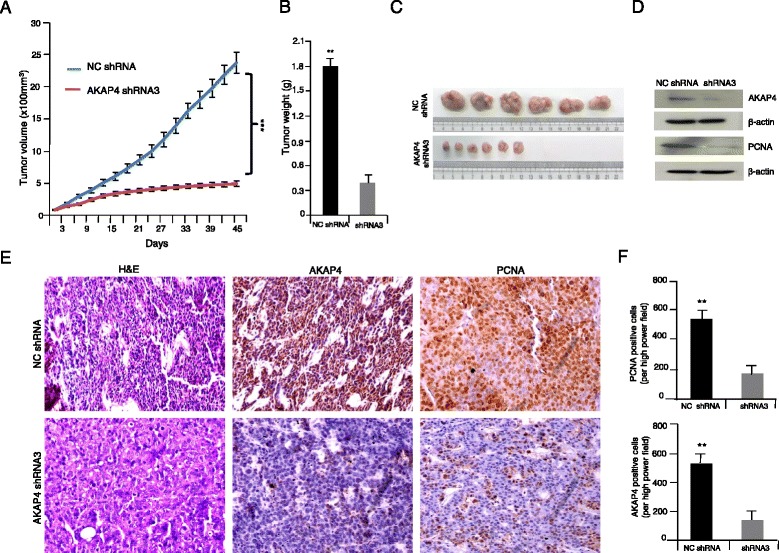
Ablation of AKAP4 protein inhibits tumor growth. **a** Graph depicts the significant difference in the tumor volume when treated with AKAP4 shRNA3 as compared to NC shRNA. **b** Histogram shows the significant reduction in tumor weight of animals treated with AKAP4 shRNA3 as compared to NC shRNA. **c** Representative images of tumor excised from the animals treated with AKAP4 shRNA3 as compared to NC shRNA. **d** Western blot analysis shows reduced expression of AKAP4 and PCNA in AKAP4 shRNA3 treated mice as compared to NC shRNA treated mice. β-actin was used as a loading control. **e** Immunohistochemical analysis on the serial sections of tumor reveals reduced expression of AKAP4 (middle panel) and PCNA (right panel) in AKAP4 shRNA3 treated mice as compared to NC shRNA treated mice. The left panel shows the cytostructure of tumour by H&E staining. **f** Histograph depicts significant reduction in AKAP4 and PCNA expression in AKAP4 shRNA3 treated mice as compared to NC shRNA treated mice. **P < 0.001, ***P < 0.0001. Original magnification x200, objective x20

To further validate our observations on malignant properties of colorectal cancer, following *AKAP4* gene silencing, we investigated the role of AKAP4 in *in-vivo* colorectal xenograft mouse model. Our studies showed a significant decrease (*p* < 0.0001) in tumor growth in AKAP4 shRNA3 treated mice as compared to NC shRNA treated mice as shown in Fig. 5. Tumor volume, weight and size were significantly reduced as shown respectively in Fig. 5a, b and c. The xenograft tumors were excised post 49 days and were subjected to Western blotting and IHC for AKAP4 and PCNA protein expression. Western blot analysis revealed the down regulation of AKAP4 and PCNA protein in AKAP4 shRNA3 treated tumor lysates as compared to NC shRNA treated tumor lysates (Fig. 5d). Further, our IHC studies demonstrated a significant reduction of 70.74 % in PCNA and 74.8 % reduction in the AKAP4 protein expression in AKAP4 shRNA3 treated mice as compared to NC shRNA treated mice (Fig. 5e and f).

**Fig. 6:**
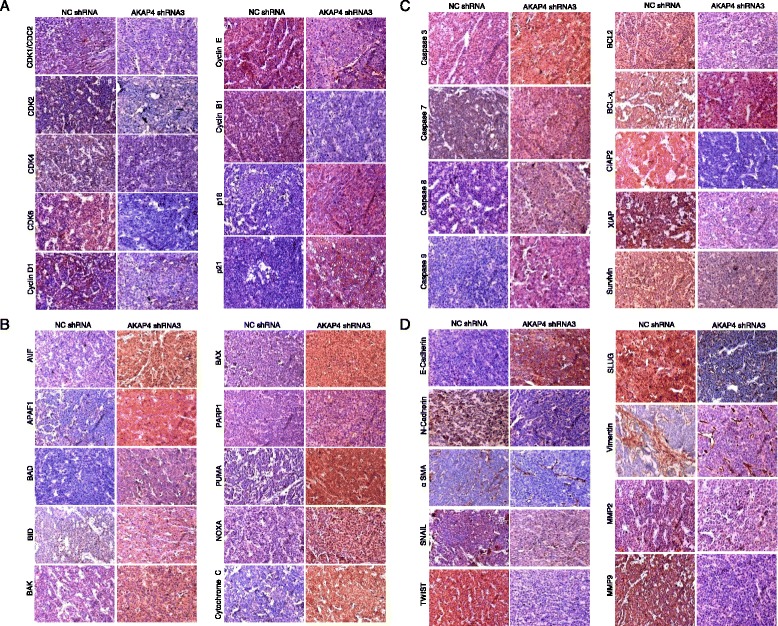
Immunohistochemical analysis of various molecules involved in cell cycle, apoptosis and EMT. **a** Representative micrographs depicts reduced immuno-reactivity of CDK1, CDK2, CDK4, CDK6, Cyclin D1, Cyclin E,Cyclin B1, and increased immuno-reactivity of p16 and p21 in AKAP4 shRNA3 treated mice as compared to NC shRNA treated mice. **b** Representative photomicrographs show increased immuno-reactivity of AIF, APAF1, BAD, BID, BAK, BAX, PARP1, PUMA, NOXA, cyt-C in AKAP4 shRNA3 treated mice as compared to NC shRNA treated mice. **c** Representative photomicrographs show increased immuno-reactivity of Caspase 3, Caspase 7, Caspase 8 and Caspase 9 and decreased immuno-reactivity of BCL2, Bcl-x_L_, cIAP2, XIAP, Survivin in AKAP4 shRNA3 treated mice as compared to NC shRNA treated mice. **d** Representative micrographs showing increased immuno-reactivity of E-cadherin and reduced immuno-reactivity of N-cadherin, α-SMA, SNAIL, TWIST, Vimentin, MMP2, MMP9 in AKAP4 shRNA3 treated mice as compared to NC shRNA treated mice. Original magnification x200, objective x20

We were intrigued by our Western blot results analysis which showed the effects of ablation of AKAP4 protein in COLO 205 and HCT 116 on various signaling molecules Figs. 2b, 3b and 4b. Therefore, we next validated the various molecules by IHC in excised tumor sections of mice treated with AKAP4 shRNA3 or NC shRNA. Our IHC findings were in perfect agreement with the Western blot results (Fig. 6). As expected, there was a down regulation of cellular proliferation molecules CDK1, CDK2, CDK4, CDK6, Cyclin D1, Cyclin E and Cyclin B1 and upregulation of p16 and p21 in the xenograft tumor tissues by IHC (Fig. 6a). Further, there was up regulation of proapoptotic (AIF APAF1, BAD, BID, BAK, BAX, PARP1, PUMA, NOXA and cyt-C (Fig. 6b) and Caspase 3, Caspase 7, Caspase 8, Caspase 9 (Fig. 6c), and down regulation of anti-apoptotic molecules (BCL2, Bcl-x_L_, cIAP2, XIAP and Survivin (Fig. 6c) in animals treated with AKAP4 shRNA3 target. The xenograft tissues sections also revealed down regulation of EMT molecules such as N-cadherin, αSMA, SNAIL, TWIST, SLUG, Vimentin, along with invasion molecules MMP2 and MMP9, whereas expression of epithelial marker E-cadherin was up-regulated (Fig. 6d). Thus, our *in vivo* findings supported our *in vitro* results indicating that AKAP4 may have role in tumor growth.

## Discussion

CRC is the third leading cause of mortality in men and women worldwide [1]. Most of the cancer-related deaths in CRC patients are as a result of early spread of cancer cells or due to reoccurrence post-surgical interventions [15]. Alterations in some key regulatory molecules involved in cell cycle, apoptosis and EMT pathways have been proposed in the initiation of carcinogenesis [16]. In this context, efforts are being made to identify and characterize tumor associated molecules for development of therapeutic targets for cancer treatment. A unique class of tumor associated antigens called cancer testis (CT) antigens has been reported in various malignancies and have been shown to be associated with tumor growth and metastasis [4]. Only few CT antigens with abundant expression, namely sperm associated antigen (SPAG9) and AKAP4 have been shown to be associated with CRC [5, 10]. In this study, we examined the involvement of AKAP4 in various malignant properties at phenotype and molecular level of cancer cells. Plasmid-based driven gene silencing approach was employed to study the role of AKAP4 in different pathways contributing in various malignant properties of CRC cells in culture and *in vivo* human xenograft mouse model.

The molecular events involved in cell cycle regulation are altered during onset of carcinogenesis and tumor growth. Especially, deregulation of the CDK-Cyclin complexes result in uncontrolled cellular proliferation [17]. Our study has put forth an evidence for the first time wherein ablation of AKAP4 gene expression in CRC cells resulted in alteration of key molecules involved in various cell cycle phases. At molecular level Cyclin D1, Cyclin E and Cyclin B1 with its partners CDK1, CDK2, CDK4 and CDK6 were found to be down-regulated. Our finding was supported by a recent study [12] which showed that ablating dual specificity phosphatase 21 (DUSP21) CT antigens down regulated Cyclin D1 and Cyclin E leading to cell cycle arrest and senescence [12]. Our data also revealed up regulation of Cyclin dependent kinase inhibitors (CKIs)- p16, p21 and Rb. Since cell cycle arrest may result in senescence, in this context we investigated senescence status of cells which showed up-regulation of putative marker, DCR2. Interestingly, SEM images also validated the flattened and elongated shape of cells following AKAP4 ablation. It is important to mention here that none of the earlier studies so far have reported such phenotypic changes at SEM level.

Chemotherapy treatment causes toxicity and also effects normal somatic tissue as well. In this regard, CT antigens may be an ideal target for cancer therapy. Since, CT antigens have restricted expression in testis and various malignancies [4], these may be used for immunotherapeutic target which may not cause any side effect on normal tissue [18]. In this context, a recent study on CT antigen MAGE-A3 with a limited number of patients revealed that postoperative MAGE-A3 immunization proved to be feasible with minimal toxicity [19]. In present investigation, we assessed the involvement of AKAP4 in cascades of various pathways contributing towards the malignant properties of cancer cells which may shed light on AKAP4 as a novel therapeutic target. We observed that ablation of AKAP4 resulted in the up-regulation of pro-apoptotic molecules such as AIF, APAF1, BAD, BID, BAK, BAX, cleaved PARP1, PUMA, NOXA, cyt-C, Caspase 3, Caspase 7, Caspase 8 and Caspase 9. Further, we also found the down regulation of anti-apoptotic molecules BCL-2, Bcl-x_L,_ cIAP2, XIAP, Axin2 and Survivin in the AKAP4-depleted CRC cells indicating that AKAP4 may be potential therapeutic target in cancer management.

EMT is an important process which supports the cancer cell migration by altering various molecular events which involve mesenchymal-epithelial transition (MET). Interestingly, ablation of AKAP4 resulted in down regulation of pro-EMT molecules including N-cadherin, P-cadherin, α-SMA, SLUG, SNAIL, TWIST, Vimentin, MMP2, MMP3 and MMP9 protein. Cell migration and invasion potential and colony forming ability were also significantly reduced due to AKAP4 down regulation. We further assessed and validated our *in vitro* results in a colorectal cancer xenograft mouse model. It is noteworthy that AKAP4 knockdown markedly inhibited the tumor growth with reduced AKAP4 and PCNA expression. Thus, AKAP4 may be used as therapeutic target for cancer treatment. Ours is the first study reporting validation by IHC of various molecules involved in cell cycle regulation, senescence, apoptosis and EMT in colon cancer xenograft model.

## Conclusion

In conclusion, the present study shows that AKAP4 is over expressed in CRC cell lines. Ablation of AKAP4 apparently has multiple effects at molecular level in various malignant properties of the cancer cells including reduction in colony formation ability, expression of EMT molecules and growth arrest of cells (senescence). We strongly believe that AKAP4 may be used as a potential therapeutic target for the development of better CRC treatment management.

## Additional file

Additional file 1: Figure S1.
**A**) Quantitative PCR: Histogram depicts the qPCR results showing knockdown efficiency of various shRNA targets against *AKAP4* gene in COLO 205 and HCT 116 cells. **B**) Colony formation assay: Representative images show the difference in number of colonies formed when COLO 205 and HCT 116 cells were transfected with NC shRNA and AKAP4 shRNA3. (PPTX 154 kb)
