# Supplementary material for: A-kinase anchor protein 4 (AKAP4) a promising therapeutic target of colorectal cancer
Source: J Exp Clin Cancer Res. 2015 Nov 21;34:142. doi: 10.1186/s13046-015-0258-y (PMC4654903; doi:10.1186/s13046-015-0258-y)
Supplement: Additional file 1: Figure S1. — A) Quantitative PCR: Histogram depicts the qPCR results showing knockdown efficiency of various shRNA targets against AKAP4 gene in COLO 205 and HCT 116 cells. B) Colony formation assay: Representative images show the difference in number of colonies formed when COLO 205 and HCT 116 cells were transfected with NC shRNA and AKAP4 shRNA3. (PPTX 154 kb) [file 13046_2015_258_MOESM1_ESM.pptx]

## Slide 1
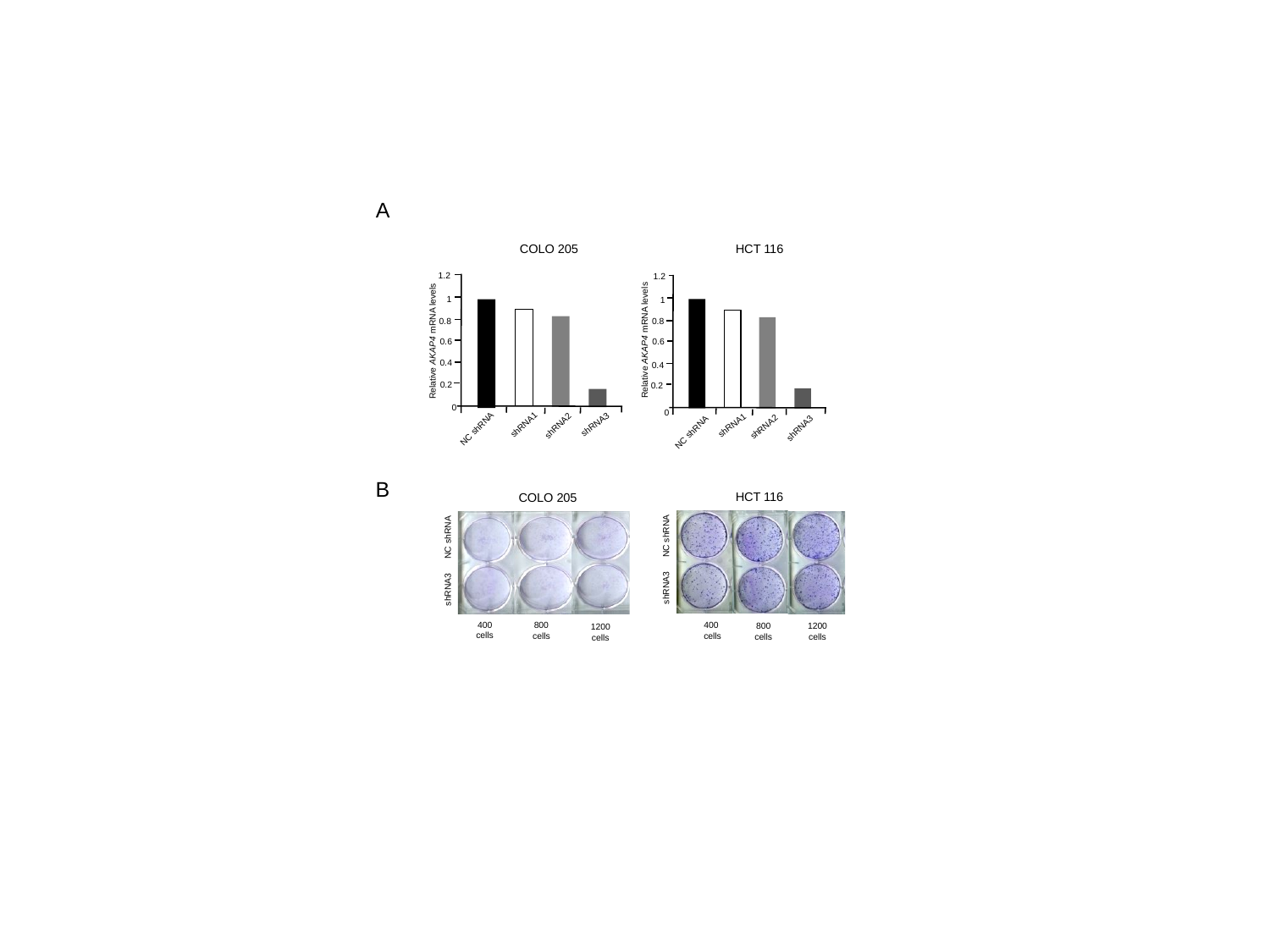

A
COLO 205
1.2
1
0.8
0.6
0.4
0.2
0
Relative AKAP4 mRNA levels
shRNA1
shRNA3
shRNA2
NC shRNA
HCT 116
1.2
1
0.8
0.6
0.4
0.2
0
Relative AKAP4 mRNA levels
shRNA1
shRNA2
shRNA3
NC shRNA
B
HCT 116
COLO 205
400
cells
1200 cells
800 cells
NC shRNA
 shRNA3
 shRNA3
NC shRNA
400 cells
800 cells
1200 cells
